# Flavonoid-Coated Gold Nanoparticles as Efficient Antibiotics against Gram-Negative Bacteria—Evidence from In Silico-Supported In Vitro Studies

**DOI:** 10.3390/antibiotics10080968

**Published:** 2021-08-12

**Authors:** Hani A. Alhadrami, Raha Orfali, Ahmed A. Hamed, Mohammed M Ghoneim, Hossam M. Hassan, Ahmed S. I. Hassane, Mostafa E. Rateb, Ahmed M. Sayed, Noha M. Gamaleldin

**Affiliations:** 1 Department of Medical Laboratory Technology, Faculty of Applied Medical Sciences, King Abdulaziz University, Jeddah 21589, Saudi Arabia; hanialhadrami@kau.edu.sa; 2 Molecular Diagnostic Lab, King Abdulaziz University Hospital, King Abdulaziz University, Jeddah 21589, Saudi Arabia; 3 Special Infectious Agent Unit, King Fahd Medical Research Centre, King Abdulaziz University, Jeddah 21589, Saudi Arabia; 4 Department of Pharmacognosy, College of Pharmacy, King Saud University, Riyadh 11495, Saudi Arabia; rorfali@ksu.edu.sa; 5 National Research Centre, Microbial Chemistry Department, 33 El-Buhouth Street, Dokki, Giza P.O. Box 12622, Egypt; ahmedshalbio@gmail.com; 6 Department of Pharmacy Practice, College of Pharmacy, AlMaarefa University, Riyadh 13713, Saudi Arabia; mghoneim@mcst.edu.sa; 7 Department of Pharmacognosy, Faculty of Pharmacy, Nahda University, Beni-Suef 62513, Egypt; hossam.mokhtar@nub.edu.eg; 8 Department of Pharmacognosy, Faculty of Pharmacy, Beni-Suef University, Beni-Suef 62513, Egypt; 9 School of Computing, Engineering & Physical Sciences, University of the West of Scotland, Paisley PA1 2BE, UK; Ahmedsayed.hassane@nhs.scot (A.S.I.H.); mostafa.rateb@uws.ac.uk (M.E.R.); 10 Aberdeen Royal Infirmary, Foresterhill Health Campus, Foresterhill Road, Aberdeen AB25 2NZ, UK; 11 Department of Microbiology, Faculty of Pharmacy, The British University in Egypt (BUE), Cairo 11837, Egypt

**Keywords:** gold nanoparticles, flavonoids, Gram-negative bacteria, DNA gyrase, in silico

## Abstract

Flavonoids are a class of bioactive plant-derived natural products that exhibit a broad range of biological activities, including antibacterial ones. Their inhibitory activity toward Gram-positive bacterial was found to be superior to that against Gram-negative ones. In the present study, a number of flavonoid-coated gold nanoparticles (GNPs) were designed to enhance the antibacterial effects of chrysin, kaempferol, and quercetin against a number of Gram-negative bacteria. The prepared GNPs were able to conjugate to these three flavonoids with conjugation efficiency ranging from 41% to 80%. Additionally, they were able to exert an enhanced antibacterial activity in comparison with the free flavonoids and the unconjugated GNPs. Quercetin-coated GNPs were the most active nano-conjugates and were able to penetrate the cell wall of *E. coli*. A number of in silico experiments were carried out to explain the conjugation efficiency and the antibacterial mechanisms of these flavonoids as follows: (i) these flavonoids can efficiently bind to the glutathione linker on the surface of GNPs via H-bonding; (ii) these flavonoids, particularly quercetin, were able to increase the bacterial membrane rigidity, and hence decrease its functionality; (iii) these flavonoids can inhibit *E. coli*’s DNA gyrase (Gyr-B) with IC_50_ values ranging from 0.9 to 3.9 µM. In conclusion, these bioactive flavonoid-based GNPs are considered to be very promising antibiotic candidates for further development and evaluation.

## 1. Introduction

One of the major drug delivery challenges is drug selectivity to avoid the potential side effect and reduce the cytotoxic effects of the therapeutic agent [1]. This selectivity plays a significant role in increasing drug efficacy, reducing the drug dosage and frequency, and controlling the release of the therapeutic agent [2]. On the other hand, using large-sized materials for drug delivery faces many challenges, starting from in vivo instability, poor solubility, poor bioavailability, and poor absorption in the body to issues with target-specific delivery [1]. Therefore, searching for a new drug delivery system is crucial for solving these critical issues. In this regard, nanotechnology can provide a variety of solutions. Beside its unlimited applications in construction material, electronics, food production, agriculture, catalysis, and energy production, it can provide unique applications and solutions for many medical and health-based issues [3,4].

Nanobiotechnology is a promising area of nanotechnology that utilizes nano-scale materials in different aspects of biology [5]. Recently, nanomaterials have gained considerable attention from scientists due to their potential applications in medicine and drug formulations [6]. With their nano size, which ranges between 1 and 100 nm, nanomaterials have gained increasing attention in many medical fields such as biosensors, microarray tests, microfluidics, tissue engineering and drug delivery [7]. Furthermore, their chemical and physical properties make them an excellent choice as a drug delivery system when compared with other larger-scale counterparts usually used for drug delivery [8]. Their high surface-area-to-volume ratio increases their affinity for small molecules and facilitates their uptake across the cell membrane [4]. Beside their unique structures, nanoparticles display magnetic, electrical, and biological properties which allow them to be good candidates for a delivery system for encapsulating drugs and deliver them more precisely with a controlled release to target tissues [4,8]. Selection of suitable nanomaterials for drug delivery is based on the drug’s physiochemical features [1].

Among all the noble metal nanoparticles, gold has gained great attention due to its unique properties, such as good conductivity, chemical stability, catalytic properties, and biological activity, including antibacterial, antifungal, antiviral and anti-inflammatory activities. It can also be used in a variety of applications, ranging from medical applications (photothermal and radiation therapy, photodynamic therapeutics, biosensors, and X-ray imagery) to food industry, material science, chemistry, and physics [9,10,11].

As a part of the continuing investigation into safe and effective antibacterial agents from natural products, a number of non-glycosylated flavonoids have been tested for their growth inhibitory activity against some Gram-negative bacteria (*Escherichia coli*, *Pseudomonas aeruginosa*, *Proteus vulgaris* and *Klebsiella pneumonia*). This class of compounds has been found to exert antibacterial activity via multiple mechanisms: (i) altering the fluidity of the bacterial membranes (i.e., increasing their rigidity), an effect that was found to be associated with bacterial growth inhibition [12]; (ii) inhibiting DNA gyrase and, in turn, bacterial DNA supercoiling [13]; and (iii) inhibiting the bacterial penicillin-binding proteins (PBPs) and thus inhibiting bacterial cell wall biosynthesis [14].

Combining some of these bioactive flavonoids with biocompatible metallic nanoparticles can improve their pharmacokinetic properties and maximize their antibacterial efficacy. Accordingly, in this investigation, a number of novel flavonoid–gold nano-conjugates were designed and their antibacterial potential against Gram-negative bacteria was explored. Additionally, a number of in silico analyses (e.g., molecular docking and dynamics) were utilized to obtain some insight into the molecular structure of these newly prepared nano-conjugates and the mode of action of their flavonoid components. The results of the present investigation can provide a good starting point to develop flavonoid-based antimicrobial nanomaterials in the future.

## 2. Results and Discussion

### 2.1. Screening of Flavonoids against Gram-Negative Bacteria

**Figure 1:**
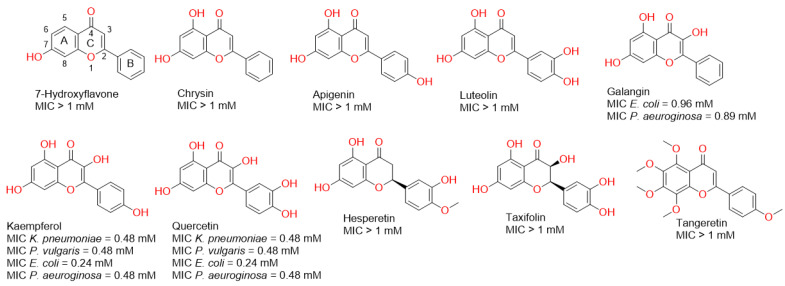
Structures of the tested flavonoids in the present study along with the antibacterial activity of each one against a number of Gram-negative bacteria.

Firstly, we evaluated the antibacterial potential of a number of flavonoids against some pathogenic Gram-negative bacteria. As shown in Figure 1, some of the tested flavonoids showed weak millimolar activity and some of them were inactive up to 1 mM. However, in our previous report, we found that some of these flavonoids (e.g., chrysin and apigenin) were far more active against Gram-positive bacteria (e.g., *Staphylococcus aureus*), where their minimum inhibitory concentrations (MICs) were in the micromolar range [14]. These significant differences in the antibacterial activity could be attributed to the Gram-negative bacterial outer membrane, which usually acts as a natural protective barrier preventing the passage of unfavourable compounds [15].

In the present study, all the flavonoids that showed growth inhibitory activity against the tested bacteria (MIC < 1 mM) shared the presence of a hydroxyl group at C-3. Additionally, the hydroxylation at Ring B correlated with enhanced activity (Figure 1). These observations indicated the most important structural elements in the scaffold of this class of compounds may develop more potent antibacterial derivatives in the future.

Our strategy in this investigation was to enhance the antibacterial activity of this class of natural products against Gram-negative bacteria via conjugating them to gold nanoparticles (GNPs). Hence, these bioactive molecules can benefit from the unique properties of the metallic nanoparticles (MNPs): (i) MNPs could act as a very good carrier for these bioactive flavonoids and could easily pass the bacterial outer membrane [16,17,18]; (ii) MNPs have the ability to concentrate bioactive molecules on their surfaces and hence maximize their activity (i.e., polyvalent effects) [19,20,21,22,23].

Accordingly, we chose the most active flavonoids (i.e., kaempferol and quercetin) against all tested Gram-negative bacteria together with chrysin, which was the most active flavonoid against the Gram-positive bacteria *S. aureus* [14], to be coated on GNPs, and subsequently tested if the new nano-conjugates could exert enhanced growth inhibitory activity towards Gram-negative bacteria. In parallel, we aimed to perform a number of in silico experiments (docking, molecular dynamic simulations, and free energy calculations) to explain the mode of conjugation of these flavonoids and their probable antibacterial mode of actions.

### 2.2. Gold Nanoparticle Preparation and Conjugation with Flavonoids

**Figure 2:**
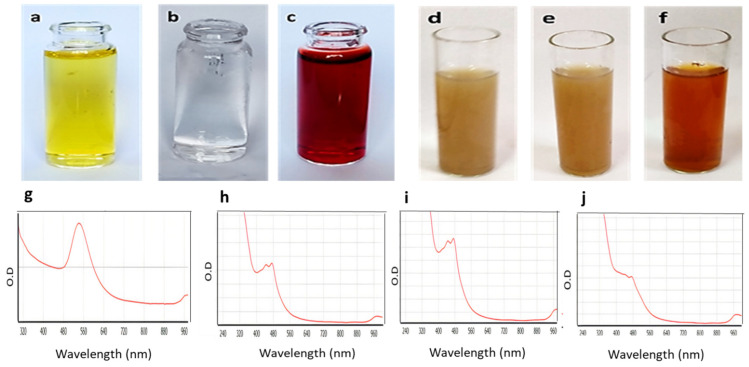
The upper panel shows the gold nanoparticles’ preparation steps: (**a**) HAuCl_4_·3H_2_O only, (**b**) HAuCl_4_·3H_2_O + GSH at pH 8, (**c**) formation of gold after the addition of NaBH_4_, and formation of gold nano-conjugates, (**d**) GNP-kaempferol, (**e**) GNP-chrysin, and (**f**) GNP-quercetin. The lower panels show (**g**) the UV-VIS spectra of the GNPs, (**h**) the UV-VIS spectra of GNP-kaempferol, (**i**) the UV-VIS spectra of GNP-chrysin, and (**j**) the UV-VIS spectra of GNP-quercetin.

Gold nanoparticles (GNPs) were prepared via chemical synthesis using reduced L-glutathione (GSH). The synthesis process was mediated by the formation of a covalent bond between the gold nanoparticles’ surfaces in the HAuCl_4_.3H_2_O compound and the cysteine thiolate of GSH. This binding led to the aggregation of GNPs on GSH molecules and, by the addition of NaBH_4_ at pH8, the formation of a ruby red colour indicated the formation of GNPs (Figure 2). GSH is a hydrophilic tripeptide, and thus coating GNPs with such molecules makes them more stable and more water-soluble. Additionally, the GSH’s carboxylate terminals are a good binder for a variety of molecules, including flavonoids, where they are able to form extensive H-bond networks with such polyhydroxylated molecules, making them a very good carrier of such bioactive chemicals. Being a natural substrate for bacteria, GSH may facilitate the entry of the whole conjugate (i.e., GSH-GNPs) inside bacterial cells [24,25].

Accordingly, the freshly prepared GSH-GNPs were incubated with each selected flavonoid to allow their binding with the GSH’s carboxylate groups; in turn, they became coated on the GSH-coated GNPs. The efficiency of conjugation of each flavonoid (in %) was found to be 80%, 71%, and 41% for quercetin, kaempferol, and chrysin, respectively. These results indicated that the degree of a flavonoid’s hydroxylation correlates well to its binding efficiency with the prepared GSH-coated GNPs.

### 2.3. UV-Vis Spectroscopy

To ensure that the GNPs were formed and successfully conjugated to each selected flavonoid, the UV-visible absorbance of the prepared GSH-GNPs and those coated with flavonoids was measured. As shown in Figure 2 (lower panel), the absorption spectrum of the free GNPs showed a λ_max_ at ~540 nm. Such UV absorbance was due to the surface plasmon excitation of the very small GNPs. The flavonoid-coated GNPs showed a considerable shift in their λ_max_ (~480 nm) in comparison with the uncoated ones (~530 nm), indicating successful coating of the flavonoids on the GNPs and, in turn, altering the plasmon resonance absorption band characteristic of GNPs.

### 2.4. Fourier Transform Infrared Spectroscopy Analysis (FTIR)

Fourier transform infrared spectroscopy (FTIR) is an essential technique to detect different functional groups via measuring the infrared spectra of emission, absorption, and photoconductivity of the materials. The FTIR spectrum is usually between 4000 and 400 cm^−1^. Supplementary Figure S1a–d presents the FTIR spectra of the GNPs, GNP-kaempferol, GNP-chrysin, and GNP-quercetin. The FTIR spectra of the prepared GNPs display the presence of the OH group at 3367.70 cm^−1^, which indicates the presence of alcohol. The C=O was measured at 1577.50 cm^−1^, while the band at 13.8732 cm^−1^ refers to C-C-C stretching. The C-O group appeared at a wavelength of 1279.66 to 1069.89 cm^−1^. These results were in good agreement with those of Jiang et al. (2007) and Sulaiman et al. (2020) [25,26]. The peaks that appeared after the conjugation of GNPs with kaempferol, chrysin, and quercetin were different in their intensities and shapes, and this might be attributed to the reactions that occurred between each flavonoid and the GNP-GSH. In general, a change in the band absorption intensities means that a physical change has occurred, while an increase in the band intensities means a change in the morphology and chemical composition of that band. The FTIR spectra for flavonoid-coated GNPs showed that the hydroxyl group (O-H) stretching vibration appears at 3399.10, 3402.80, and 3346.14, respectively, while the bands appearing at 2841.57, 2844.74, and 2834.72 cm^−1^ reflected the alkane (C-H) stretching vibration. Additionally, the carbonyl (C=O) stretch vibration was displayed at 1663.90, 1663.77, and 1582.04 cm^−1^. The aromatic (C=C) stretch bands were observed at 1521.42, 1521.42, and 1416.94 cm^−1^, respectively. The aromatic C-O stretch was observed at bands 1381.27 to 1131.00 cm^−1^. All the peaks characterizing flavonoids were observed. These results were in good agreement with those of Kiroula et al. (2016) and Sulaiman et al. (2020) [26,27].

### 2.5. X-ray Powder Diffraction (XRD)

**Figure 3:**
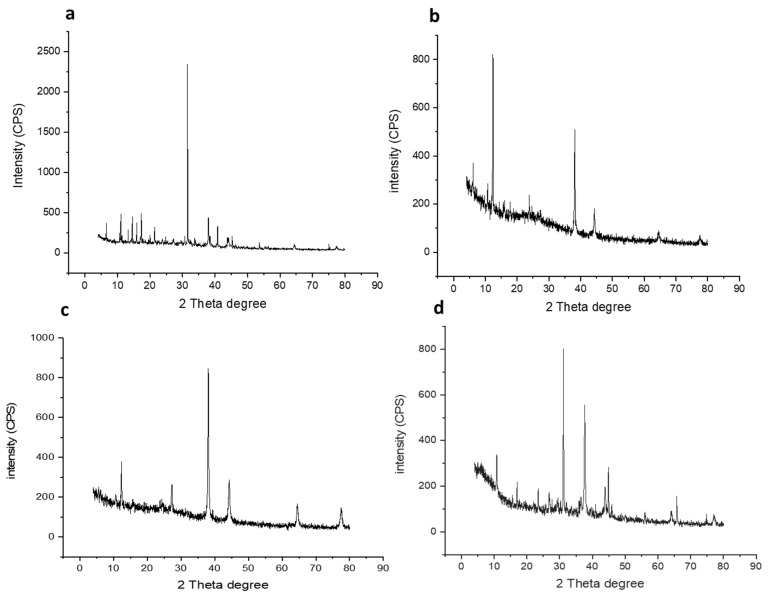
X-ray diffraction analyses for (**a**) prepared GNPs, (**b**) GNP-kaempferol, (**c)** GNP-chrysin, and (**d**) GNP-quercetin.

One of the most important techniques used to study structural properties is X-ray powder diffraction (XRD). The diffraction pattern of the prepared GNPs and GNP-coated flavonoids (i.e., GNP-quercetin, GNP-chrysin, and GNP-quercetin) were analysed. As depicted in Figure 3a–d, the most important characteristic peaks of the Au phase appeared at 38.10°, 44.5°, 64.06° and 77.45°, accredited to the crystallographic planes (111), (200), (220), and (311), respectively. Additionally, the intensity of GSH appeared at 31.53°. Whereas, in flavonoid-coated GNPs (Figure 3), they displayed characteristic peaks for quercetin, kaempferol, and chrysin at 23°, 38°, 44°, 64°, and 77°.

### 2.6. Electron Microscopy

**Figure 4:**
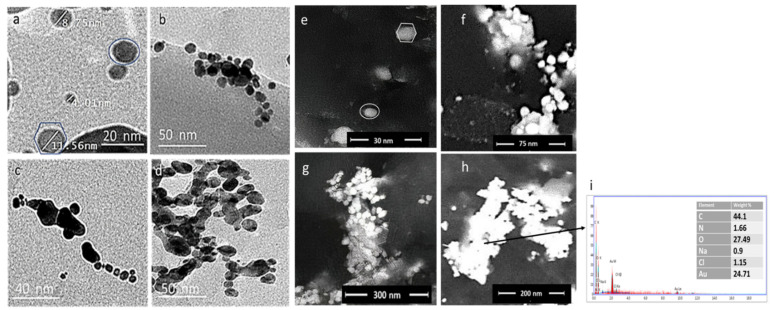
TEM micrographs for (**a**) prepared GNPs (**b**) GNP-kaempferol (**c**) GNP-chrysin, and (**d**) GNP-quercetin and FESEM micrographs for (**e**) prepared GNPs, (**f**) GNP-kaempferol, (**j**) GNP-chrysin, and (**h**) GNP-quercetin. (**i**) Elemental analysis by energy dispersive X-ray (EDX) analysis for GNP-quercetin.

The particle size and morphology of the prepared GNPs, GNP-kaempferol, GNP-chrysin, and GNP-quercetin were determined via transmission electron microscopy (TEM) (Figure 4a–d) and field emission scanning electron microscopy (FESEM) (Figure 4e–h). The average particle size of the GNPs is about ~4.10 ± 2 to 35 ± 2 nm with monodisperse spherical and hexagonal prism-like shapes (Figure 4a). The TEM and FESEM micrographs of GNP-kaempferol, GNP-chrysin, and GNP-quercetin showed spherical and homogenous structures (Figure 4b–d,f–h).

### 2.7. Energy Dispersive X-ray Spectroscopy (EDX)

To measure the elemental composition of the prepared GNPs, GNP-kaempferol, GNP-chrysin, and GNP-quercetin, the samples were tested using EDX. The EDX of the GNPs confirmed the presence of Au with different percentages in each sample up to 24.71% in GNP-quercetin (Figure 4i), with the presence of carbon and oxygen in sufficient percentages to confirm the loading of kaempferol, chrysin, and quercetin on GNPs.

### 2.8. In Vitro Investigation

#### 2.8.1. In Vitro Antibacterial Activity

The antibacterial activity of the GNP conjugates was measured against a panel of clinical isolates comprising Gram-negative bacteria (*E. coli*, *P. aeruginosa*, *K. pneumonia* and *P. vulgaris*). The minimum inhibitory concentrations (MIC) were determined by the micro-dilution method using 96-well plates. The MIC results showed that the activity of the tested GNPs (GNP-kaempferol, GNP-chrysin, and GNP-quercetin) was quite diverse. The results showed that GNP-quercetin was generally the most active against all tested Gram-negative bacteria, with pronounced activity against *E. coli*, *P. aeruginosa* and *P. vulgaris* (MIC = 30 μg/mL) and *K. pneumonia* (MIC = 60 μg/mL). On the other hand, the prepared GNP-kaempferol exhibited good antibacterial properties against *E. coli* and *P. vulgaris* with MICs of 60 and 30 μg/mL, respectively, while it displayed weak activity against *P. aeruginosa* (MIC = 240 μg/mL) and *K. pneumonia* (MIC = 120 μg/mL). The GNP-chrysin showed good inhibitory activity against *E. coli* (MIC = 60 μg/mL) and weak activity against the rest of the tested microbes (MIC > 240 μg/mL). Ciprofloxacin was used as a positive control.

#### 2.8.2. GNP-Induced Disruption of Bacterial Cell Membranes

**Figure 5:**
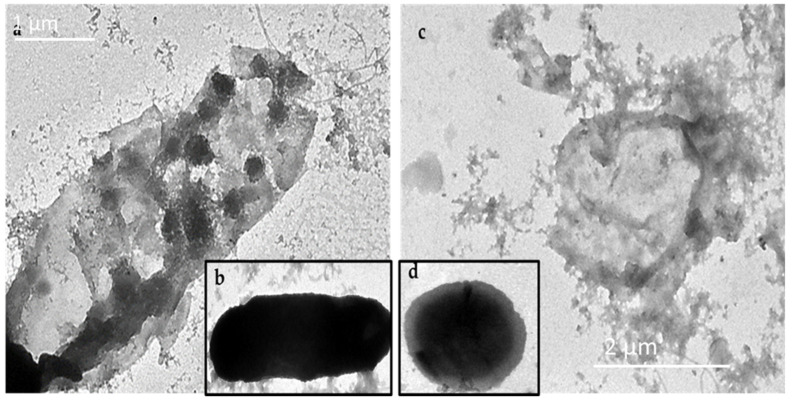
(**a**) *E. coli* + GNP-quercetin, (**b**) *E. coli* without treatment, (**c**) *P. aeruginosa* + GNP-quercetin, and (**d**) *P. aeruginosa* without treatment.

To explore the antibacterial mechanism of these GNPs, the GNP-quercetin conjugate was selected, since it showed the best antibacterial activities (Table 1). After treating suspensions of *E. coli* or *P. aeruginosa* (5.0 × 105 cfu mL^−1^) with GNP-quercetin conjugate at a final concentration of 20 µg mL^−1^, incubated at 37 °C overnight, the antibiotic effect of the GNP-quercetin conjugate on the membrane morphology and nucleic acid leakage of *E. coli* and *P. aeruginosa* was visualized using transmission electron microscopy (TEM). The TEM micrograph clearly displayed the broken membranes of the treated *E. coli* and *P. aeruginosa* (Figure 5a–c) with the clear appearance of GNP-quercetin inside the bacterial cell. Figure 5b,d presents the two bacterial strains without treatment. Free uncoated GSH-GNPs, in contrast, were not able to have similar effects on *E. coli*, in which the membrane integrity remained intact and no particles were seen inside the bacterial cells.

#### 2.8.3. In Vitro DNA Gyrase-B Inhibition

Quercetin, kaempferol, and chrysin have been reported to produce negative supercoiling of the bacterial DNA via the inhibition of DNA gyrase [13]. However, the exact mode of action of these molecules is still elusive. DNA gyrase consists of two subunits: subunit A and B (Gyr-A and -B, respectively). Gyr-A is the subunit that binds to DNA and relaxes its positive supercoils. Additionally, it is targeted by fluoroquinolone antibiotics [28]. On the other hand, Gyr-B is responsible for obtaining the required energy of this process via hydrolysing one molecule of ATP [14].

**Figure 6:**
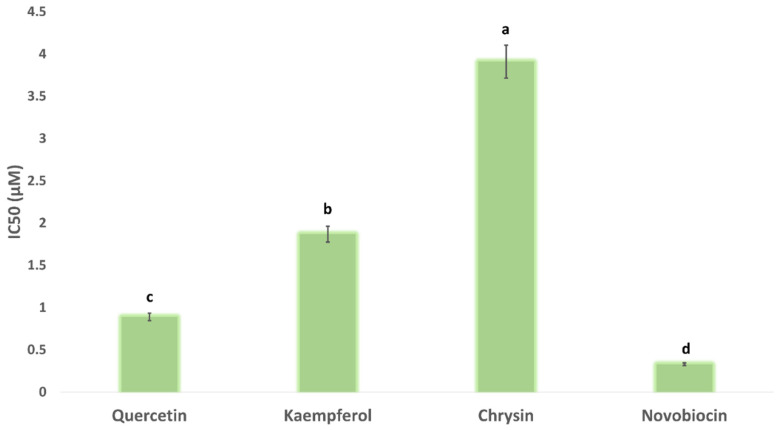
Gyr-B inhibitory activity of quercetin, kaempferol, and chrysin expressed as IC_50_. Bars represent standard errors. Different letters indicate significant differences from repeated-experiments (*n* = 3) ANOVA (Hotelling’s *T*^2^, *p* < 0.05).

Upon docking these flavonoids (i.e., quercetin, kaempferol, and chrysin) against the binding site of each subunit (Gyr-A and B), they achieved significantly higher docking scores with Gyr-B (−9.5, −9.5, −9.3 kcal/mol, respectively) than with Gyr-A (−5.1, −4.9, and −5.2, respectively). Accordingly, we tested these flavonoids for their Gyr-B inhibitory activity. As shown in Figure 6, the three flavonoids produced micromolar inhibition of Gyr-B’s activity. Quercetin was the most potent inhibitor, with an IC_50_ value of 0.89 ± 0.1 µM, while chrysin was the least potent (IC_50_ = 3.91 ± 0.2 µM), where the flavonoids’ degree of hydroxylation apparently correlated to their Gyr-B inhibitory activity.

### 2.9. In Silico Investigation

To gain more insight into the antibacterial mode of action of these flavonoids, we carried out a number of in silico experiments (e.g., docking, molecular dynamic simulations (MDS), and binding free energy calculations).

**Figure 7:**
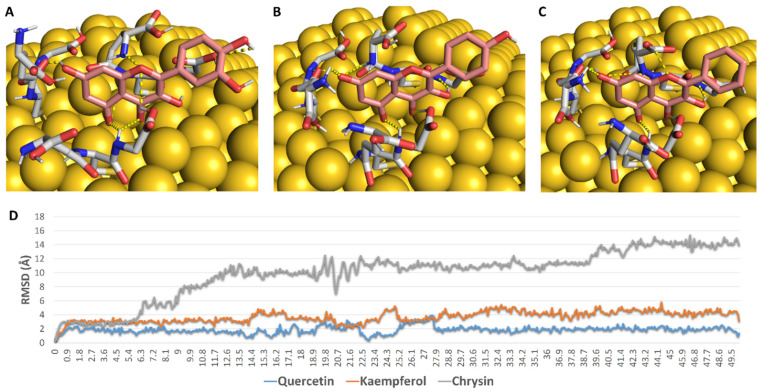
(**A**–**C**): Binding mode of quercetin, kaempferol, and chrysin with GSH molecules covalently linked to gold molecules on the surface of GNP. (**D**): RMSDs of these flavonoids during the course of 50 ns MDS.

First, we aimed to study the interaction between each flavonoid and the GSH interface of 5 nm GNP. To do so, we constructed a model consisting of GNP (5 nm) linked to three GSH molecules via covalent bonds between their SH groups and three Au atoms at the surface (Figure 7). Subsequently, we docked each flavonoid against this constructed GSH coating. As shown in Figure 7, the three flavonoids took almost the same orientation between the three GSH molecules, establishing a network of H-bonds between their OH groups and a number of polar moieties in the GSH molecules (e.g., their carboxylate and amide groups) (Figure 7). During the course of MDS (50 ns), these extensive H-bonds kept the binding orientations of both quercetin and kaempferol almost unchanged (RMSD~2.1 Å and 3.8 Å, respectively), while chrysin was far less stable (RMSD~10.3 Å). These binding behaviours appeared to be linked to the Ring B hydroxyl groups that were involved in multiple H-bonds with GSH’s carboxylate arms during the course of MDS. Accordingly, the two flavonoids with a hydroxyl group or groups at Ring B were more stable in their interactions with GSH in comparison with chrysin, which had no hydroxyl groups at Ring B. The results of MDS were in good accordance with those of the percent efficiency of conjugation, where the flavonoids with the highest stability with GNP-coated GSH (quercetin and kaempferol) were found to achieve the highest binding efficiency (80% and 71%, respectively), while chrysin, which was significantly less stable, achieved less binding efficiency (41%).

Second, we aimed to study the effect of each flavonoid on the bacterial outer membrane (OM). Previously, it was found that this class of natural products (i.e., flavonoids) exert their antibacterial activity against *E. coli* via increasing the rigidity of its outer membrane (OM) [12,29]. In this study, we found a very good correlation between the growth inhibitory activity of these flavonoids and their effect on the rigidity of a membrane model composed of dipalmitoylphosphatidylcholine (DPPC) and 1,2-dipalmitoyl-sn-glycero-3-phosphoglycerol (DPPG) (used as a representative of the bacterial membrane), where the increase in this membrane’s rigidity was accompanied by increased antibacterial activity.

**Figure 8:**
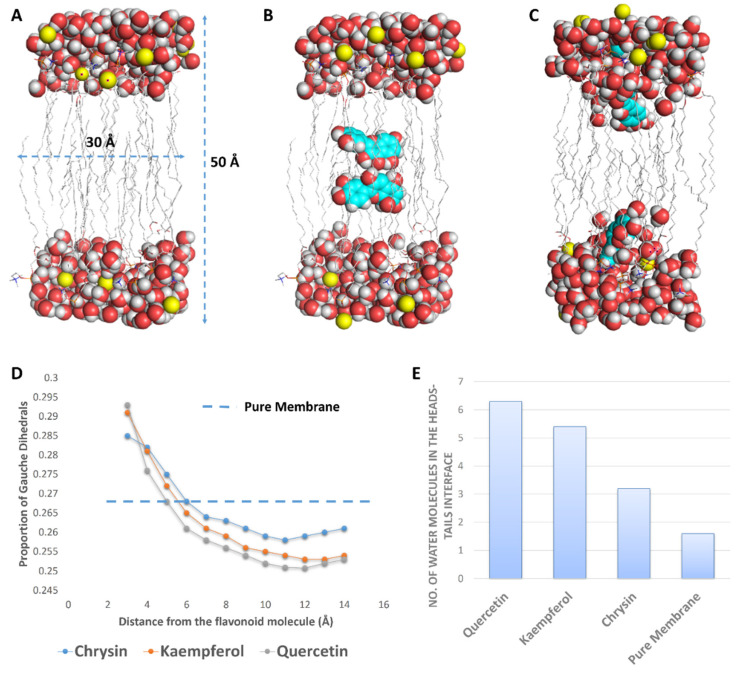
(**A**) The lipid bilayer system used for the MDS. (**B**) The lipid bilayer system with two molecules of quercetin at the beginning of a 100 ns MDS. (**C**) The lipid bilayer system at the end of 100 ns of MDS, where each quercetin molecule was stabilized at the head–tail interface. (**D**) Proportion of gauche dihedrals on lipid tail carbon atoms as a function of the lipid’s distance from each flavonoid molecule. (**E**) Number of water molecules per lipid molecule at the head–tail interface.

To study this effect at a molecular level, we performed a number of MDS experiments on lipid bilayer systems consisting of DPPC and DPPG (1:1) with two molecules of each flavonoid inserted inside the core of the lipid bilayer (Figure 8). When each flavonoid was added to the simulations, they spontaneously directed to the head–tail interface, where they partitioned themselves and stayed stable until the end of the simulations. Additionally, each flavonoid molecule attracted a number of water molecules thanks to their hydroxyl groups; hence, the water density at the hydrophilic–hydrophobic interface increased (Figure 8). This increase in water molecules at the head–tail interface was proportional to the degree of hydroxylation, and thus quercetin and kaempferol attracted the highest number of water molecules (~4 molecules), while chrysin attracted the fewest.

Hydrated membranes are usually more fluid than less hydrated ones and have higher numbers of gauche defects (distortion in the lipid alkyl chains) [30,31,32]. Accordingly, polyhydroxylated flavonoids (e.g., quercetin) were able to draw water from the surrounding environment at the hydrophilic–hydrophobic interface, producing a local increase in the water density and, in turn, a local increase in the membrane’s fluidity (a high proportion of gauche defects). At the same time, this effect led eventually to a dehydrated membrane with much less fluidity (i.e., increased rigidity) and a low proportion of gauche defects. Quercetin produced the highest proportion of local gauche defects (0.293 at 3 Å from quercetin) but, at the same time, drew the largest number of water molecules (~6 molecules), producing the highest dehydration effect and the lowest proportion of gauche defects (~0.245 at 6 to 10 Å away from quercetin). The proportion of gauche defects in the lipid bilayer without flavonoids was ~0.268, while this proportion, in general, was significantly lower in the presence of each flavonoid (Figure 8). These results were in very good accordance with that reported for caffeine, which was found to increase the rigidity of 1-palmitoyl-2-oleoyl-sn-glycero-3-phosphocholin (POPC) membranes via the same mechanism [32]. Being able to concentrate flavonoids on their surfaces, our prepared GNPs in this study had the ability to significantly increase the effect of each flavonoid on the bacterial OM (i.e., polyvalent effects) and hence maximize their antibacterial potential.

**Figure 9:**
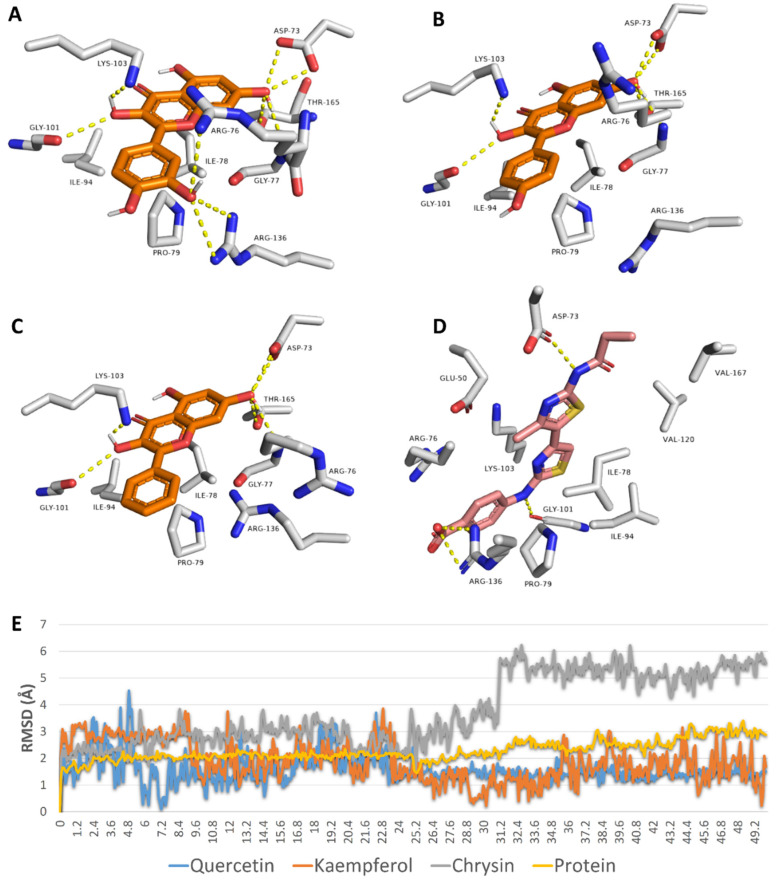
Docking poses of quercetin, kaempferol, and chrysin inside the active site of *E. coli*’s Gyr-B (**A**–**C**, respectively) alongside the binding pose of the co-crystalized inhibitor (**D**). RMSDs of quercetin, kaempferol, and chrysin inside the active site of *E. coli*’s Gyr-B during 50 ns of MDS (**E**).

Third, we studied the binding mode of each flavonoid inside the active site of *E. coli*’s Gyr-B to rationalize their inhibitory effects. Quercetin, kaempferol, and chrysin achieved convergent docking scores with Gyr-B’s active site (−9.5, −9.5, and −9.3 kcal/mol). Additionally, they took an orientation and exhibited molecular interactions inside the active site that were very close to that of the co-crystalized inhibitor (Figure 9). Being rich in hydroxyl groups, quercetin was able to establish a network of H-bonds with ASP-73, GLY-77, GLY-101, LYS-103, ARG-136, and THR-165. Furthermore, it established a number of hydrophobic interactions with ILE-78, PRO-79, and ILE-94. Both kaempferol and chrysin exhibited the same interactions, except for the H-bond with ARG-136. To further validate these docking results, all of these binding poses were subjected to 50 ns MDS. Both quercetin and kaempferol were able to keep their binding orientation with low deviations (RMSD~1.5 Å), while chrysin deviated more from its docking pose, with an average RMSD of 4.4 Å. The three flavonoids achieved almost identical binding free energies (Δ*G*s) (i.e., −8.5, −8.5, and −8.2 kcal/mol). All of these in silico analyses explained the low micromolar inhibition of the three flavonoids against *E. coli*’s Gyr-B. The present in vitro and in silico results correlated well with those of Wu and co-workers on flavonoids against DNA gyrase [13].

## 3. Materials and Methods

### 3.1. Chemicals

HAuCl_4_·3H_2_O and reduced L-glutathione (GSH) were purchased from Sigma-Aldrich Chemical Co. (St. Louis, MO, USA), lysogeny broth (LB broth) and (NaOH) were purchased from Merck (Mainz, Germany), while sodium borohydride from Strem Chemicals, Inc. (Newburyport, MA, USA). All other chemicals and reagents were of analytical grade. Regarding the flavonoids used in this study, all of them were isolated from their plant sources [14], except for quercetin, apigenin, and hesperetin, which were purchased from Alfa Aesar, Massachusetts, USA, and Sigma-Aldrich, Saint Louis, USA. All of these flavonoids were of acceptable purity (i.e., >98%).

### 3.2. Microorganisms

*Pseudomonas aeruginosa* ATCC10145, *Escherichia coli* ATCC25955, *Proteus vulgaris* ATTC7829, and *Klebsiella pneumonia* ATCCBAA-1705 were obtained from the Faculty of Medicine, Al-Azhar University, Egypt.

### 3.3. Gold Nanoparticle Preparation

Gold nanoparticles were prepared as described by Wu et al. (2014) and Sulaiman et al. (2020) [26,33], 5 mL of a tetrachloroauric acid aqueous solution (0.025 M) was added to 50 mL of 0.019 M of a reduced L-glutathione (GSH) aqueous solution, and the mixture was vigorously stirred for 30 min. The pH of the mixture was adjusted to 8 using NaOH (0.1 M). To this mixture, freshly prepared aqueous NaBH_4_ (2 mg·mL^−1^) was added dropwise under vigorous stirring until the formation of a ruby red colour. To remove the excess GSH and other salts, the GNPs were centrifuged for 3 h at 5000 rpm. After the centrifugation, the supernatant was removed and the gold nanoparticles were dispersed in double-distilled water; the centrifugation was repeated 2 times to obtain clean GNPs that were kept in the dark at 4 °C for 14 days (aggregations were detected after 17 days of storage). Freshly prepared nanoparticles were then conjugated with the flavonoids (i.e., kaempferol, chrysin, and quercetin).

### 3.4. Conjugation of Kaempferol, Chrysin, and Quercetin with Gold Nanoparticles

Conjugation of kaempferol, chrysin, and quercetin with the prepared GNPs was carried out according to [26]. Here, 1 mL of the prepared GNPs (3.5 × 10^13^; GNPs/mL) was mixed with 1 mL of the flavonoid solution (i.e., kaempferol, chrysin, or quercetin) (500 μg mL^−1^) and stirred overnight at room temperature. After preparation, the conjugates (GNP-kaempferol, GNP-chrysin, and GNP-quercetin) were centrifuged for 1 h at 10,000 rpm to remove any excess of the drug.

The freshly conjugated nanoparticles were used for the antibacterial assay immediately after their preparation. They were also kept in the dark at 0 °C. These conjugated nanoparticles can be used after storage for 10 days (they produced the same results in the antibacterial assay and no aggregation was observed up to 10 days of storage).

### 3.5. Characterization of Prepared Nanoparticles

#### 3.5.1. UV-Vis Spectroscopy Measurements

The synthesis of GNPs was primarily visualized through the change in colour. The conversion of Au^3+^ to Au^0^ was monitored by measuring the UV-vis spectra within wavelengths ranging from 220 nm to 1000 nm in the mixture over time. The UV-vis spectra of the solutions were measured in 96-well flat polystyrene plates by using a SPECTROstar nano absorbance plate reader (BMG LABTECH).

#### 3.5.2. X-ray Diffraction (XRD) Studies

To study the X-ray diffraction pattern of the prepared GNPs and flavonoid–GNPs conjugates, samples were drop-coated onto a glass material. The XRD analysis was performed using a PANalytical X’pert PRO X-ray diffractometer (The Netherlands) with Cu Ka1 radiation under an operating voltage and tubing current of about 40 kV and 30 mA, respectively. The diffracted patterns were recorded at 2θ from 10° to 80° at the scanning speed of 0.02°/min.

#### 3.5.3. Fourier-Transform Infrared Spectroscopy (FTIR)

The ATR-FTIR spectra of GNPs and flavonoid–GNPs conjugates were carried out using a Broker vertex 80 v in the range of 4000–400 cm^−1^ with a resolution of 4 cm^−1^, according to Brock-Neely (1957).

#### 3.5.4. Transmission Electron Microscopy Analysis (TEM)

Transmission electron microscopy (TEM) was used to measure the size and obtain the morphology of the prepared GNPs and flavonoid–GNPs conjugates. Preparation of the samples was carried out by placing 2–4 µL of the prepared solution on carbon-coated copper grids. The thin film that formed was air-dried at room temperature and observed using a Philips 10 Technai with an accelerating voltage of about 180 keV with a wavelength (λ) of 0.0251 Å. The average size of the prepared nanoparticles was measured using Image J software.

#### 3.5.5. Scanning Electron Microscope (SEM)

Scanning electron microscopy (SEM) was carried out using a field emission scanning electron microscope (FE-SEM) (Quanta FEG-250, Netherlands) with the acceleration voltage at 20 kV, attached to EDX (energy dispersive x-ray analysis) to perform the elemental analysis.

### 3.6. Determination of the Antimicrobial Activity of Flavonoids, GNPs, and Flavonoid–GNPs Conjugates

The antibacterial activity of the flavonoids (Figure 1), the prepared GNPs and the flavonoid–GNPs conjugates was acquired against four Gram-negative bacteria: *Escherichia coli* ATCC25955, *Pseudomonas aeruginosa* ATCC10145, *Proteus vulgaris* ATTC7829, and *Klebsiella pneumonia* ATCCBAA-1705. The minimal inhibitory concentration was assessed in 96-well flat polystyrene plates by addition of 80 µL of lysogeny broth (LB broth) in each well, followed by the addition of 10 µL of a bacterial culture suspension (log phase), then 10 µL of the test material (the flavonoids, the prepared GNPs, or the flavonoid–GNPs) was added. The final concentrations of the mixture were 960, 480, 240, 120, 60, 30, 15, 7.5, 3.25, 1.62 and 0.81 μg/mL. The plates were incubated at 37 °C for 24 h. After incubation, the positive antibacterial activity of the flavonoids, the prepared GNPs, and the flavonoid–GNPs was observed as clearance in the wells, which was confirmed by measuring the absorbance after about 20 h at OD_600_ in a Spectrostar Nano Microplate Reader (BMG LABTECH GmbH, Allmendgrun, Germany). The MIC experiments were carried out three times and gave the same results each time. Ciprofloxacin was used as a positive control.

### 3.7. In Vitro Enzyme Assay

Gyrase subunit B (Gyr-B) in vitro inhibitory activity was carried out using the Inspiralis assay kit (Inspiralis, UK) according to previous protocols [34,35]. The procedure of this in vitro assay is described in detail in Supplementary File 1.

### 3.8. In Silico Investigation

#### 3.8.1. Molecular Docking

Before the docking experiments the gold–GSH model was prepared. First, we used the online nanomaterial modeller software Charm GUI (https://charmm-gui.org/?doc=input/nanomaterial, accessed on 20 June 2021) [36] to construct a 6 nm spherical gold nanoparticle model. Subsequently, we linked three molecules of glutathione (GSH) to three surface gold atoms via covalent bonds (i.e., between the SH group of GSH and the gold atom). We kept a distance of 7 Å between each GSH molecule to avoid steric clashes between them, particularly during molecular dynamic simulations. After that, we enclosed the three GSH molecules in a grid box for docking experiments using the Auto Dock tools 1.5.4 program [37]. Chrysin, kaempferol, and quercetin were docked on this predetermined grid box using AutoDock 4 software [37]. The top scores were then selected and visualized using Pymol software [38].

#### 3.8.2. Molecular Dynamic Simulations

All molecular dynamics experiments were carried out by Desmond v. 2.2 and NAMD software [39,40] using OPLS and Charmm27 force fields, respectively. Further experimental details can be found in the Supplemenatry Table S1.

### 3.9. Statistical Analysis

Three independent experiments were carried out to provide the results in the present investigation, expressed as the means ± SE (*n* = 3). Statistical significance was determined by ANOVA (*p* < 0.05).

## 4. Conclusions

Free uncoated GNPs exhibited moderate antibacterial properties, while flavonoids exhibited weak inhibitory activity against Gram-negative bacteria. The outer membrane may be the shield of Gram-negative bacteria against this type of natural product because several flavonoids have previously shown very good potential against Gram-positive strains. Concentrating flavonoids on the surface of GNPs could maximize their inhibitory potential against Gram-negative bacteria. Accordingly, we formulated novel flavonoid-coated gold nano-conjugates to detect their antibacterial properties. We used GSH as a linker between the surface of GNPs and the flavonoids. To select the best flavonoids to be coated on the surface of GSH-GNPs, we screened a number of flavonoid derivatives against four common pathogenic Gram-negative bacteria. Quercetin, kaempferol, and chrysin were found to be the most active candidates; however, their activity was expressed in the millimolar range. Coating GSH-GNPs with these flavonoids led to the preparation of efficient growth-inhibitory nano-conjugates against Gram-negative bacteria. Quercetin-coated gold nano-conjugate was the most potent one and was able to penetrate the cell wall of *E. coli*. These nano-conjugates were able to fight Gram-negative bacteria via two proposed mechanisms: (i) increasing the bacterial membrane’s rigidity, hence decreasing its functionality; and (ii) targeting the bacterial subunit B of DNA gyrase (Gyr-B). By conducting a series of in silico and in vitro experiments, we found that quercetin was the best flavonoid in terms of its binding efficiency to the GSH-GNPs, increasing the membrane rigidity and inhibiting Gyr-B. In conclusion, flavonoid-coated GNPs are considered to be promising antibiotic candidates for further development and evaluation.

## Figures and Tables

**Table 1:** Antibacterial activity of the gold nano-conjugates against Gram-negative bacteria.

Tested Compound	MIC (μg/mL)			
	*E. coli*	*P. aeruginosa*	*K. pneumonia*	*P. vulgaris*
Free GNPs	120	240	120	120
GNP-quercetin	30	30	60	30
GNP-kaempferol	60	240	120	30
GNP-chrysin	60	>240	>240	>240
Cip	1	1	1	2

Cip: ciprofloxacin. MIC values were the same in three independent experiments.

## Data Availability

All relevant data are contained within the article.

## Supplementary Materials

The following are available online at https://www.mdpi.com/article/10.3390/antibiotics10080968/s1. Figure S1: Fourier-transform infrared spectroscopy for (a) prepared GNPs, (b) GNP-kaempferol, (c) GNP-chrysin, and (d) GNP-quercetin. Table S1: MIC absorbance data. File 1: In Vitro Enzyme Assay.
