# Supplementary material for: Flavonoid-Coated Gold Nanoparticles as Efficient Antibiotics against Gram-Negative Bacteria—Evidence from In Silico-Supported In Vitro Studies
Source: Antibiotics (Basel). 2021 Aug 12;10(8):968. doi: 10.3390/antibiotics10080968 (PMC8389009; doi:10.3390/antibiotics10080968)
Supplement: Supplementary file 1 [file antibiotics-10-00968-s001.zip › antibiotics-1304266-supplementary.pdf]

Supplementary Material

Flavonoids-Coated Gold Nanoparticles as Efficient Antibiotics against Gram-Negative Bacteria –Evidence from *In Silico*-Supported *In vitro* Studies.

Hani A. Alhadrami[1,2,3,†] Raha Orfali,[4,†] Ahmed A. Hamed,[5] Mohammed M. Ghoneim,[6] Hossam M. Hassan,[7,8] Ahmed S. I. Hassane,[9,10] Mostafa E. Rateb,[9] Ahmed M. Sayed,[7,*] Noha M. Gamaleldin[11,*]

[1] Department of Medical Laboratory Technology, Faculty of Applied Medical Sciences, King Abdulaziz University, P. O. Box 80402 Jeddah 21589, Saudi Arabia

[2] Molecular Diagnostic Lab, King Abdulaziz University Hospital, King Abdulaziz University, P.O. BOX 80402, Jeddah 21589, Saudi Arabia

[3] Special Infectious Agent Unit, King Fahd Medical Research Centre, King Abdulaziz University, P. O. Box 80402 Jeddah 21589, Saudi Arabia

[4] Department of Pharmacognosy, College of Pharmacy, King Saud University. P. O. Box 22452, Riyadh 11495, Kingdom of Saudi Arabia

[5] Microbial Chemistry Department, National Research Centre, 33 El-Buhouth Street, P.O. Box 12622, Dokki, Giza, Egypt

[6] Department of Pharmacy Practice, College of Pharmacy, AlMaarefa University, Ad Diriyah, Riyadh 13713, Saudi Arabia.

[7] Department of Pharmacognosy, Faculty of Pharmacy, Nahda University, Beni-Suef 62513, Egypt. Ahmed.mohamed.sayed@nub.edu.eg. hossam.mokhtar@nub.edu.eg

[8]Department of Pharmacognosy, Faculty of Pharmacy, Beni-Suef University, Beni-Suef 62513, Egypt.

[9] School of Computing, Engineering & Physical Sciences, University of the West of Scotland, Paisley PA1 2BE, UK

[10] Aberdeen Royal Infirmary, Foresterhill Health Campus, Foresterhill Road, AB25 2NZ, Aberdeen, UK

[11] Department of Microbiology, Faculty of Pharmacy, The British University in Egypt (BUE), Cairo 11837, Egypt. noha.gamaleldin@bue.edu.eg

[†] Authors equally contributed to this work

* Correspondence to AMS (Ahmed.mohamed.sayed@nub.edu.eg) and NMG (noha.gamaleldin@bue.edu.eg).

Supplementary File 1: In Vitro Enzyme Assay

In vitro Gyr-B Assay

Gyrase subunit B (Gyr-B) in Vitro inhibitory activity were determined using the Inspiralis assay kit (Inspiralis®, UK) on streptavidin-coated 96-well microtiter plates (Thermo Scientific, Germany) according to the manufacturer's protocols. The assay measures the ability of the tested compounds to inhibit the ATPase activity of Gyr-B subunit. Briefly, the plates were hydrated with buffer (20 mM Tris-HCl with pH 7.6, 0.01% *w*/*v* BSA, 0.05% *v*/*v* Tween 20, 137 mM NaCl) and the biotinylated oligonucleotide was then immobilized. After the unbound oligonucleotide was washed out, the enzyme inhibitory assay was performed. The reaction volume of 30 μL in buffer (35 mM Tris × HCl with pH 7.5, 4 mM $MgCl_2$, 24 mM KCl, 2 mM DTT, 1.8 mM spermidine, 1 mM ATP, 6.5 % *w*/*v* glycerol, 0.1 mg/mL albumin) contained 1.5 U of DNA gyrase from *E. coli*, 0.75 μg of relaxed pNO1 plasmid, and 3 μL solution of the inhibitor in 10% DMSO and 0.008% Tween 20. Subsequently, the reaction solutions were incubated at 37 °C for 30 min. At the end of the reaction, the TF buffer (50 mM NaOAc with pH 5.0, 50 mM NaCl, and 50 mM $MgCl_2$) was added. After additional incubation for 30 min at room temperature, during which the biotinoligonucleotide-plasmid triplex was formed, the unbound plasmid was washed off using TF buffer (10 mM Tris HCl with pH 8.0 and 1 mM EDTA). The produced fluorescence was measured using a microplate reader (BioTek Synergy, excitation: 485 nm, emission: 535 nm, Germany).

Molecular Dynamics Simulation

Desmond v. 2.2 software was used for performing MDS experiments [1–3]. This software applies the OPLS force field. The lipid bilayer system was generated using the online modeling platform CHARMM-GUI (https://charmm-gui.org/). Thereafter, Membrane Builder was selected to construct the membrane under study. We made this membrane model consisted of DPPC and DPPG (1:1) with dimensions of 30 × 40 Å [4, 5]. The lipid bilayer structure was embedded in an orthorhombic box of TIP3P water together with 0.15 M

Na+ and Cl− ions in 20 Å solvent buffer. VMD software was used to place the flavonoids structures inside the constructed lipid bilayer model.

Afterward, the prepared systems were energy minimized and equilibrated for 10 ns. Desmond software automatically parameterizes inputted ligands during the system preparation step according to the OPLS force field. Gyr-B simulations were performed by NAMD [6], the parameters and topologies of the compounds were calculated either using the Charmm27 force field with the online software Ligand Reader and Modeler (http://www.charmm-gui. org/?doc=input/ligandrm, accessed on 16 April 2021) [7] or using the VMD plugin Force Field Toolkit (ffTK). Afterward, the generated parameters and topology files were loaded to VMD to readily read the protein–ligand complexes without errors and then conduct the simulation step.

Binding Free Energy Calculations

Binding free energy calculations (ΔG) were performed using the free energy perturbation (FEP) method [7]. This method was described in detail in the recent article by Kim and coworkers [7]. Briefly, this method calculates the binding free energy $\Delta G_{binding}$ according to the following equation: $\Delta G_{binding} = \Delta G_{Complex} - \Delta G_{Ligand}$. The value of each ΔG is estimated from a separate simulation using NAMD software. Interestingly, all input files required for simulation by NAMD can be papered by using the online website Charmm-GUI (https://charmm-gui.org/?doc=input/afes.abinding, accessed on 16 April 2021). Subsequently, we can use these files in NAMD to produce the required simulations using the FEP calculation function in NAMD. The equilibration was achieved in the NPT ensemble at 300 K and 1 atm (1.01325 bar) with Langevin piston pressure (for ”Complex” and ”Ligand”) in the presence of the TIP3P water model. Then, 10 ns FEP simulations were performed for each compound, and the last 5 ns of the free energy values was measured for the final free energy values [7]. Finally, the generated trajectories were visualized and analyzed using VMD software.

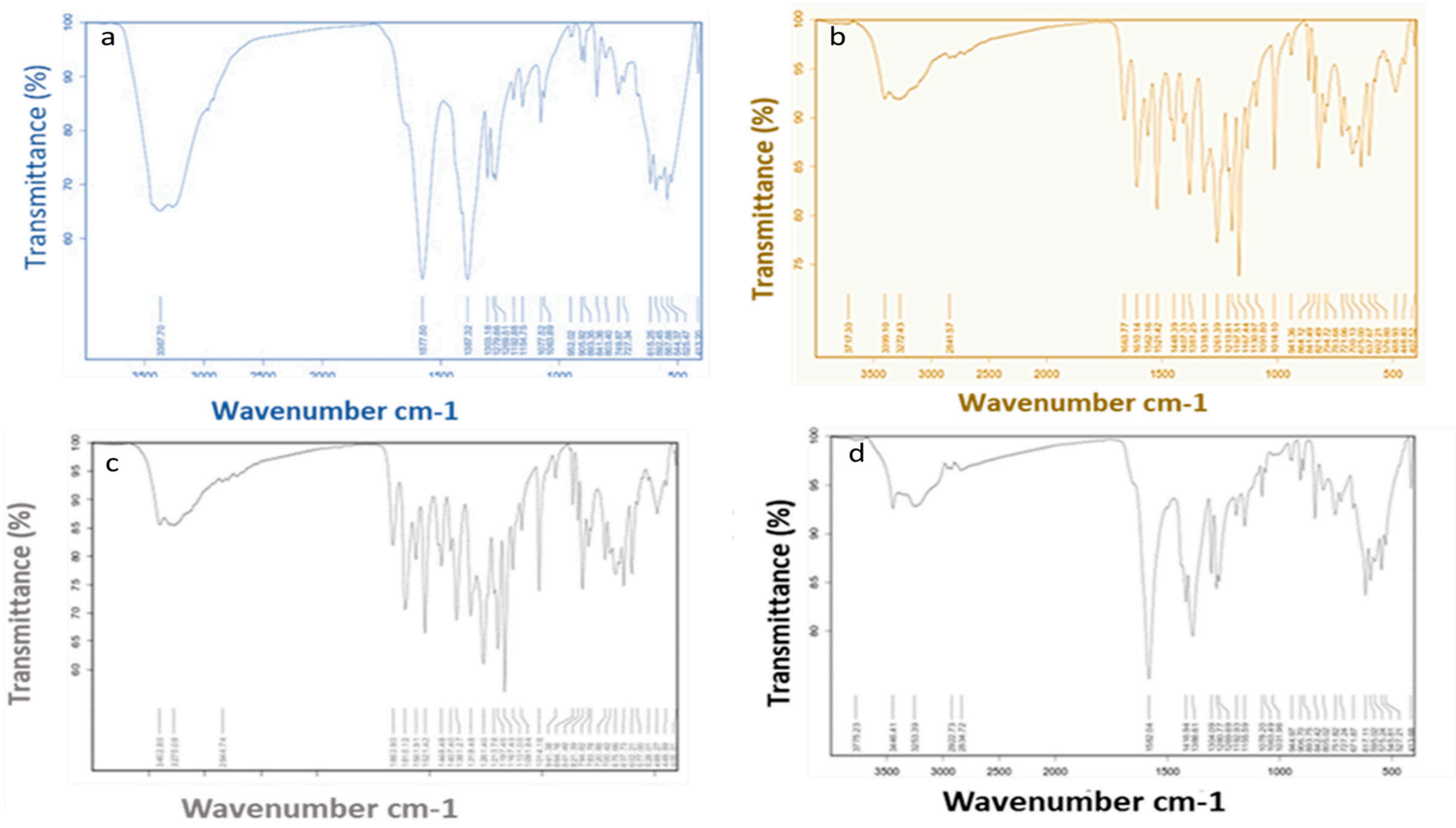

Figure S1. Fourier-transform infrared spectroscopy for (a) prepared GNPs (b) GNP- kaempferol (c) GNP-chrysin and (d) GNP-quercetin.

Table S1. MIC absorbance data

GNPs									
0.81	1.62	3.25	7.5	15	30	60	120	240	**Concentration (µg/mL)**
0.631	0.639	0.635	0.625	0.626	0.352	0.192	0.08	0.026	***E. coli***
0.611	0.601	0.605	0.621	0.614	0.355	0.123	0.072	0.05	***P. aeruginosa***
0.79	0.751	0.758	0.709	0.812	0.811	0.806	0.571	0.354	***K. pneumonia***
0.55	0.557	0.582	0.571	0.558	0.539	0.54	0.46	0.198	***P. vulgaris***

GNPs-quercetin									
0.81	1.62	3.25	7.5	15	30	60	120	240	**Concentration (µg/mL)**
0.55	0.557	0.582	0.571	0.558	0.339	0.24	0.11	0.05	***E. coli***
0.624	0.645	0.622	0.626	0.627	0.314	0.134	0.022	0.016	***P. aeruginosa***
0.832	0.822	0.825	0.835	0.822	0.819	0.421	0.251	0.15	***K. pneumonia***
0.556	0.551	0.552	0.561	0.64	0.337	0.121	0.083	0.045	***P. vulgaris***

GNPs-kaempferol									
0.81	1.62	3.25	7.5	15	30	60	120	240	**Concentration (µg/mL)**
0.652	0.614	0.632	0.6	0.603	0.606	0.424	0.201	0.035	***E. coli***
0.55	0.526	0.5	0.496	0.507	0.514	0.51	0.52	0.512	***P. aeruginosa***
0.632	0.652	0.599	0.603	0.623	0.619	0.621	0.45	0.36	***K. pneumonia***
0.512	0.51	0.51	0.502	0.514	0.512	0.35	0.15	0.065	***P. vulgaris***

GNPs-chrysin									
0.81	1.62	3.25	7.5	15	30	60	120	240	**Concentration (µg/mL)**
0.568	0.529	0.524	0.535	0.53	0.498	0.435	0.185	0.095	***E. coli***
0.555	0.535	0.545	0.523	0.516	0.551	0.521	0.502	0.541	***P. aeruginosa***
0.714	0.721	0.701	0.712	0.689	0.701	0.703	0.705	0.741	***K. pneumonia***
0.482	0.49	0.505	0.512	0.501	0.521	0.503	0.501	0.515	***P. vulgaris***

References

1. Bowers, K. J., Chow, D. E., Xu, H., Dror, R. O., Eastwood, M. P., Gregersen, B. A., ... & Shaw, D. E. (2006, November). Scalable algorithms for molecular dynamics simulations on commodity clusters. In *SC'06: Proceedings of the 2006 ACM/IEEE Conference on Supercomputing* (pp. 43-43). IEEE.

2. Release, S. 3: Desmond Molecular Dynamics System, DE Shaw Research, New York, NY, 2017; Maestro-Desmond Interoperability Tools, Schrödinger: New York, NY, USA, 2017.

3. Schrodinger LLC. Maestro, Version 9.0; Schrodinger LLC: New York, NY, USA, 2009.

4. Venable, R. M., Sodt, A. J., Rogaski, B., Rui, H., Hatcher, E., MacKerell Jr, A. D., Klauda, J. B. (2014). CHARMM all-atom additive force field for sphingomyelin: elucidation of hydrogen bonding and of positive curvature. *Biophysical journal*, *107*(1), 134-145.

5. Klauda, J. B., Venable, R. M., Freites, J. A., O'Connor, J. W., Tobias, D. J., Mondragon-Ramirez, C., ... & Pastor, R. W. (2010). Update of the CHARMM all-atom additive force field for lipids: validation on six lipid types. *The journal of physical chemistry B*, *114*(23), 7830-7843.

6. Phillips, J.C.; Braun, R.; Wang, W.; Gumbart, J.; Tajkhorshid, E.; Villa, E.; Chipot, C.; Skeel, R.D.; Kalé, L.; Schulten, K. Scalable molecular dynamics with NAMD. J. Comput. Chem. 2005, 26, 1781–1802.

7. Kim, S.; Oshima, H.; Zhang, H.; Kern, N.R.; Re, S.; Lee, J.; Rous, B.; Sugita, Y.; Jiang, W.; Im, W. CHARMM-GUI free energy calculator for absolute and relative ligand solvation and binding free energy simulations. J. Chem. Theory Comput. 2020, 16, 7207–7218.
